# Mutactimycin AP, a New Mutactimycin Isolated from an Actinobacteria from the Atacama Desert

**DOI:** 10.3390/molecules27217185

**Published:** 2022-10-24

**Authors:** Rishi Vachaspathy Astakala, Gagan Preet, Bruce F. Milne, Julius Tibyangye, Valeria Razmilic, Jean Franco Castro, Juan A. Asenjo, Barbara Andrews, Rainer Ebel, Marcel Jaspars

**Affiliations:** 1 Marine Biodiscovery Centre, Department of Chemistry, University of Aberdeen, Old Aberdeen AB24 3UE, UK; 2 CFisUC, Department of Physics, University of Coimbra, Rua Larga, 3004-516 Coimbra, Portugal; 3 Department of Medical Laboratory Sciences, Faculty of Health Sciences, Muni University, Arua P.O. Box 725, Uganda; 4 Centre for Biotechnology and Bioengineering (CeBiB), University of Chile, Beauchef 851, Santiago 8370459, Región Metropolitana, Chile; 5 Instituto de Investigaciones Agropecuarias, INIA-Quilamapu, Av. Vicente Méndez, 515, Chillán 3800062, Ñuble, Chile

**Keywords:** *Saccharothrix*, mutactimycin, anthracycline, glycoside, anthraquinone, NMR, mass spectrometry, antibiotics, ESKAPE pathogens, bovine mastitis, dairy cattle

## Abstract

Bacteria belonging to the phylum *Actinobacteria* are a very good source of antibiotics, and indeed dominate the current clinical antibiotic space. This paper reports Mutactimycin AP, a new compound belonging to an anthracycline-type family of antibiotics, isolated from a *Saccharothrix sp.* This actinobacterial strain was isolated from the rhizosphere of lupine plants growing in the extreme hyper-arid Atacama Desert. Structural characterization was carried out using electrospray ionization-mass spectrometry (ESI-MS) and NMR spectroscopy in combination with molecular modelling. The compound was tested against the ESKAPE pathogens, where it showed activity against MRSA and five strains associated with bovine mastitis, where it showed activity against *Enterococcus pseudoavium* and *Staphylycoccus Aureus subsp. Aureus*.

## 1. Introduction

Actinomycetes are an important source of antibacterial agents with approximately two-thirds of antibiotics in clinical use being isolated from this phylum of GC rich Gram-positive bacteria [1]. These bacteria can colonize the rhizosphere of plants and form a beneficial relationship with the plants by inhibiting plant pathogens [1]. *Streptomyces* bacteria are the largest genus in *Actinobacteria* and the largest producers of antibiotics in the phylum [1,2,3]. Bacterial strains isolated from the Atacama Desert are particularly interesting because of their ability to produce a variety of important compounds such as Chaxamycins [4] and Abenquines [5] and several other classes of structurally diverse compounds [6]. For this study, several actinobacterial strains were isolated from the rhizosphere of lupine plants growing in northern Chile. Among these, one strain belonging to the genus *Saccharothrix* was isolated. This genus is known to produce interesting, glycosylated compounds [7,8].

**Figure 1:**
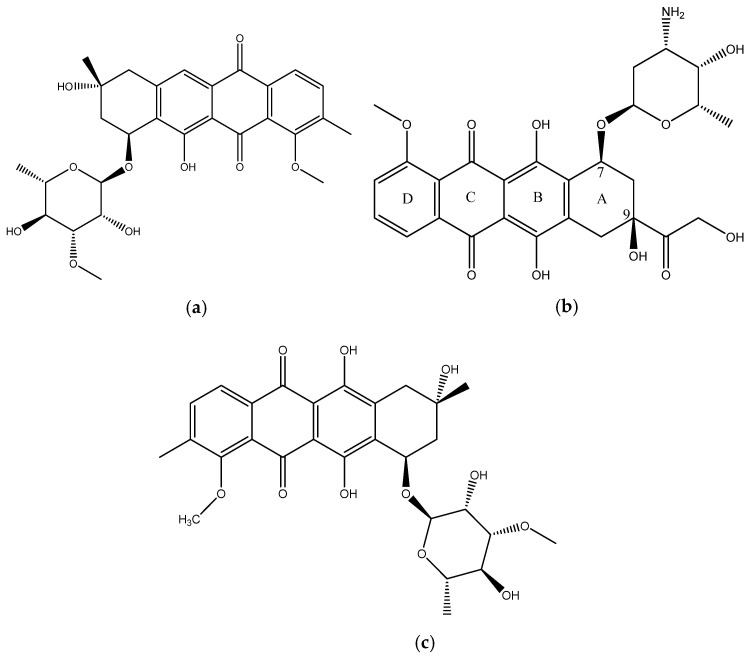
(**a**) structure of Mutactimycin AP (**1**) (**b**) structure of Doxorubicin (**2**), an early anthracycline anti-cancer drug [12] (**c**) Structure of Mutactimycin A (**3**).

In this paper we report the isolation and characterization of a new compound, mutactimycin AP (**1**) (Figure 1a) from the *Saccharothrix* strain isolated from the Atacama Desert. It belongs to a family of anthracycline-type compounds called mutactimycins [9,10] which tend to have antibacterial properties. Anthracyclines are also a broad class of highly effective anti-cancer compounds [11] which have an anthraquinone ring and an aminoglycoside as a carbohydrate side chain attached to the anthraquinone ring. The ring moiety typically tends to be tetracyclic with C7 of ring A being attached to the carbohydrate [12]. Doxorubicin (**2**) (Figure 1b), along with the other anthracyclines are a highly useful anti-cancer drug class which have been in clinical use for decades against a variety of cancers and as such only a few types of cancers are not susceptible to members of the class [13].

Mutactimycins have structures very similar to doxorubicin, the key differences being the absence of carbonylated substituents on ring A and the use of alkyl deoxy-glycosides [14] or deoxy-glycosides [15] as the carbohydrate moieties. Mutactimycins often show strong antibacterial activity typically against Gram-positive bacteria but weaker activity against gram-negative bacteria [14,15]. Examples of strong anti-bacterial activity of mutactimycins are those of mutactimycin A (**3**) (Figure 1c) and mutactimycin E against methicillin resistant *Staphylococcus aureus* [14,15]. This paper reports the structural characterization of mutactimycin AP. This paper also reports the biological activity of mutactimycin AP against one strain in the ESKAPE pathogen panel, which are a group of highly virulent nosocomial pathogens which cause life threatening and often fatal infections. The compound was also tested against five pathogens associated with bovine mastitis, a highly prevalent dairy cattle disease caused by bacterial infections, where it showed activity against two strains.

## 2. Results and Discussion

In the positive mode ESI-MS spectrum of **1**, the sodium adduct of the monomer at *m*/*z* 551.1888 (C_28_H_32_O_10_Na, D: −0.1 ppm; msigma: 17.8) and that of the dimer at *m*/*z* 1079.3908 were observed quite prominently, which is a pattern consistent with at least one other member of the family [14,15]. The ion at *m*/*z* 351.1227 indicated the [M+H]^+^ of the aglycone moiety (C_21_H_19_O_5_ D: −1.4 ppm; msigma: 5.1). The masses appearing in the spectrum suggested that the aglycone for compound **1** was different from Mutactimycin A by an oxygen atom. The UV showed strong peaks at 230 nm and 260 nm with a weaker but a broad peak starting 300 nm and ending at 500 nm with maximum intensity at 400 nm, which is unlike the other members of the family which typically show the broad peak from 350 nm to 550 nm. This hypsochromic shift in compound **1** can be explained by the lack of oxygen at position 11 on the anthraquinone ring which is present in other members of the family [14,15]. 

**Figure 2:**
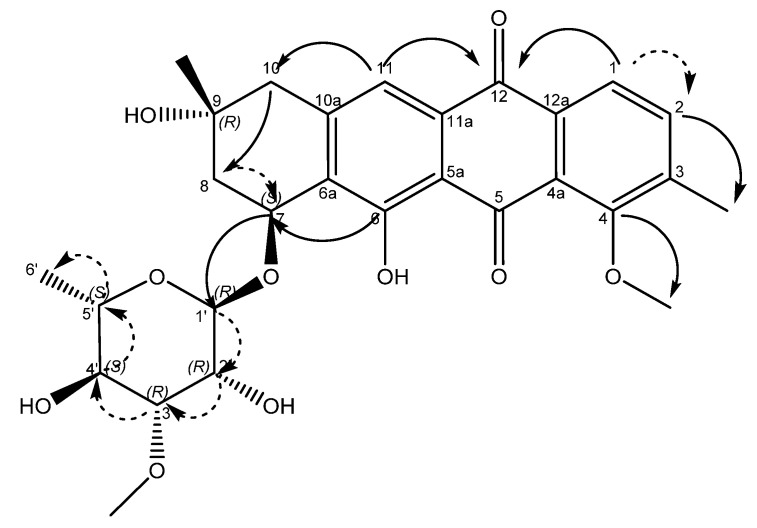
Structure of compound **1** with key HMBC correlations shown as bold arrows and COSY correlations shown as dashed arrows.

**Figure 3:**
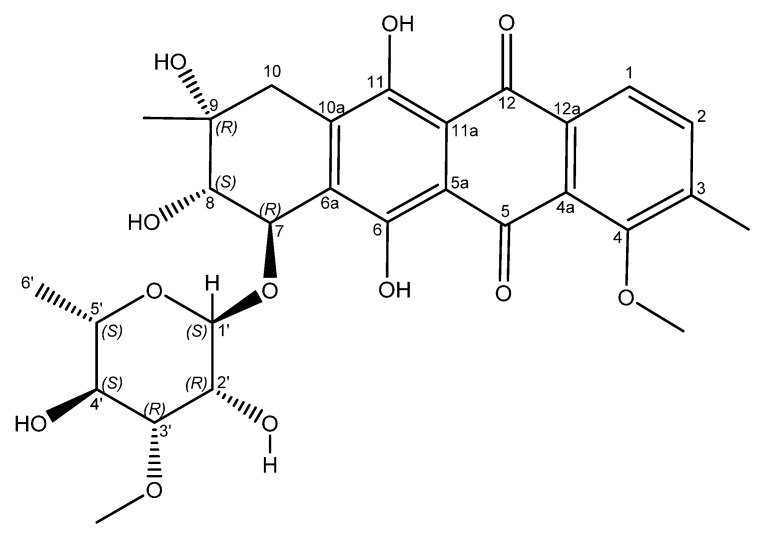
Structure of mutactimycin E (**4**).

An examination of the COSY spectrum revealed four spin systems with one of them belonging to the sugar moiety. Comparing the coupling constants of the protons and the ^13^C chemical shifts in the sugar moiety with literature values revealed that the carbohydrate moiety is α-3-*O*-methyl rhamnose [14] and is assumed to be the same absolute stereochemistry previously reported for other mutactimycins (1′R, 2′R, 3′R, 4′S, 5′S) [13]. The broad singlet at δ_H_ 5.11 (H-1′) shows an HMBC correlation with the carbon at δ_C_ 72.7 (C-7), showing that the sugar is attached at position 7. Two of the other spin systems were diastereotopic pairs at positions 8 and 10, with the final spin system belonging to the aromatic ring at the other end of the molecule. The HMBC correlation between δ_H_ 7.35 (H-11) with δ_C_ 44.3 (C-10) establishes the connection between the anthraquinone ring and the alkyl ring, whereas the HMBC correlations between H10A, H10B and C-8 along with the correlation between δ_H_ 4.89 (H-7) and δ_C_ 67.5 (C-9) establishes the structure of the rest of the alkyl ring. Similarly, HMBC correlations between δ_H_ 3.82 (H-4OMe) and δ_C_ 158.9 (C-4) and between δ_H_ 2.35 (H-3Me) and δ_C_ 137.2 (C-2) can be used to establish the positions of substituents on the benzene ring. A broad singlet at δ_H_ 13.5 establishes the presence of the phenolic proton while the HMBC correlation between H-7 and C-6 (δ 161.7) further establishes the connection between the anthraquinone ring and the alkyl ring. While the NMR data was consistent with other mutactimycins [14,15], the presence of the singlet proton at δ_H_ 7.35 (H-11), which showed an HSQC correlation at δ_C_ 122.93 and a strong HMBC correlation at δ_C_ 181.52 (C-12) and a weak HMBC correlation at δ_C_ 188.28 (C-5), indicating that it is part of the anthraquinone ring, distinguishes **1** from other members of this family as a deoxy Mutactimycin, whereas all other Mutactimycins have a hydroxy group at position 11.The overall NMR data is summarized in Table 1 and Figure 2 with annotated data provided in Supplementary Materials (Figures S1–S9). Table 1 also has compares NMR data for **1** with mutactimycin E (**4**) (Figure 3).

The stereochemistry of positions 7 and 9 was established by comparing the ROESY and NOESY spectra of **1** with previously published [14,15] NOESY/ROESY spectra and ^13^C shifts [15]. The ^13^C shifts for positions 7,8, and 9 matched those in literature [9,15], suggesting the same relative stereochemistry as previously isolated counterparts, however position 10 was found to be downfield compared to other mutactimycins possibly due to the lack of oxygen at position 11. This result led us to relying on the ROESY and NOESY data for further confirmation of the result. 

The key pieces of evidence in the ROESY spectrum used to conclude the stereochemistry were:

1. the lack of a correlation between H7 and H9Me.
2. The presence of a strong correlation between H3′ and H9Me.

**Figure 4:**
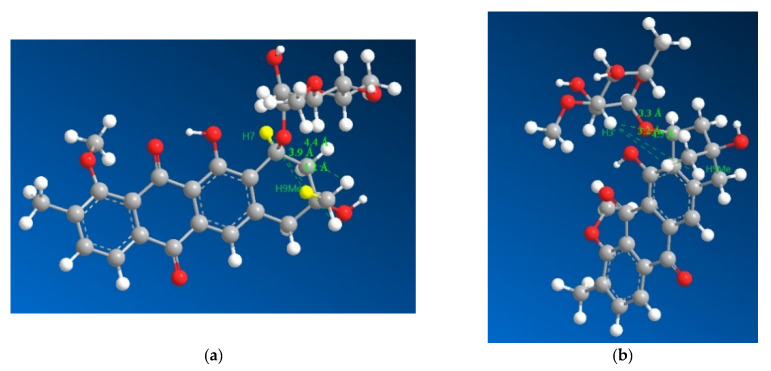
3D representations of **1** with distances between H7 and the three hydrogens of H9Me shown in (**a**) as 3.9, 4.4 and 5.2 Å with the distances between H3′ and the three hydrogens of H9Me shown in (**b**) as 3.3, 3.2 and 4.5 Å, which suggested that H7 and H9Me are on the opposite sides of the ring and that the sugar moiety folds over the ring towards the methyl group on position 9, which is consistent with previously isolated mutactimycins.

However, the result needed to be further validated owing to a reliance on negative evidence to confirm the stereochemistry. Hence, the four diastereomers possible were modelled for their lowest energy conformer after which their geometries were optimized and the distances between the hydrogens on 9Me and 7, 3′ were measured (Figure 4), which revealed that the relative stereochemistry deduced based on 13C chemical shifts (7S* and 9R*) fit into the limits for a ROESY correlation between H3′ and H9Me to appear in the spectrum with the ROESY correlation between H7 and H9Me to disappear from the spectrum. Based on the larger distances and their geometric means (Table S1) between the protons H7, H3′ and H9Me, diastereomers with 7S9S and 7R9R can be safely ruled out. Therefore, combining the data from the 13C chemical shifts and the ROESY correlations together with the previously reported absolute stereochemistry of the sugar moiety and modelling of the 7S9R and 7R9S enantiomers, it can be concluded that the 7S9R stereochemistry fits all the data best and matches the previously reported absolute stereochemistry for the mutactimycin aglycone. The specific rotation ([α]_D_^20^) was measured to be +60^°^ using methanol as the solvent. 

Compound **1** was tested against the six ESKAPE pathogens, and showed significant activity against S. aureus DSM2569 (MRSA), only with an 18 mm zone of inhibition as compared to the positive control, which was 30 µg of oxytetracycline which showed a 23 mm zone of inhibition. The MIC value observed against methicillin resistant *S. aureus* DSMZ 2569 (MRSA) was 3.125 µg/mL and MBC of 6.25 µg/mL. There is an urgent need to discover and develop novel compounds to manage life-threatening bacterial, fungal, and viral diseases because of their rising prevalence and capacity to evolve resistance to existing treatment drugs in human pathogens. These compounds need to be highly bioavailable, selective, and of low toxicity. Typically, the best substances for this purpose come from natural sources like plants and microorganisms [16].

Compound **1** was also tested against a panel of pathogens associated with bovine mastitis consisting of *Enterococcus pseudoavium* NCIMB 13084, *Escherichia coli* NCIMB 701266, *Klebsiella oxytoca* NCIMB 701361, *Staphylococcus aureus subsp. Aureus* NCIMB 701494, and *Streptococcus bovis* NCIMB 702087. Bovine Mastitis is a highly prevalent dairy cattle disease characterized by inflammation of the mammary glands caused by bacterial infection which often results in significant changes in milk produces both physical and chemical along with microbial composition [17]. It is one of the most common diseases in dairy cattle across the world. The high prevalence of the disease also leads to wastage of milk, reduction in milk quality and increased cost in treating animals, thus affecting the dairy industry’s growth and development. Of the five pathogens tested, mutactimycin AP (1) at 70 μg/mL has shown activity against *Enterococcus pseudoavium* NCIMB 13084 and *Staphylococcus aureus subsp. Aureus* NCIMB 701494 using the disc diffusion assay after which the MIC was determined. The positive control for the disc diffusion assay was 2 μg of oxalonic acid, whereas DMSO was the negative control. The results from the assays are summarized in Table 2.

With the recent increase in antibiotic resistance, there is a pressing need for research into novel antibacterial therapeutic agents that might mitigate and attenuate their pathogenicity. Natural products, especially actinomycete secondary metabolites, are an excellent pool of unrealized potential for structurally diverse antibiotics [18,19]. 

**Figure 5:**
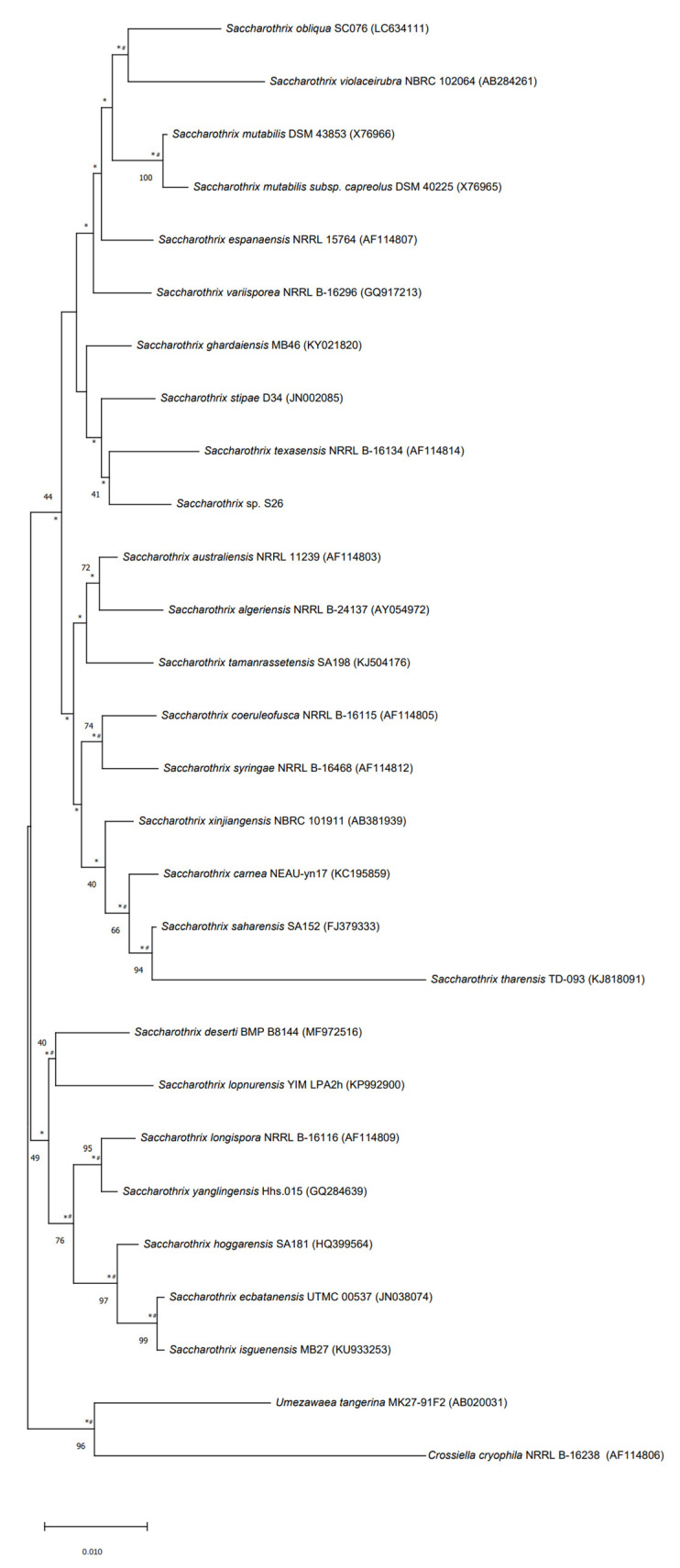
Neighbor-joining phylogenetic tree based on 16S rRNA gene sequences of strain S26 with strains belonging to *Saccharothrix* genus, and *Umezawaea tangerina* MK27-91F and *Crossiella cryophila* NRRL B-16238 used to root the tree. Asterisks and hashes indicate branches of the tree that were recovered using maximum-likelihood and maximum-parsimony methods, respectively. Numbers at the nodes indicated levels of bootstrap based on the neighbor-joining method, only values above 40% are showed. The scale bar indicates 0.010 substitutions per nucleotide position.

The strain S26 is closely related to *Saccharothrix saharensis* SA152T, with 98.91 % of similarities corresponding to 15 nucleotide differences, followed by *Saccharothrix carnea* NEAU-yn17T and *Saccharothrix stipae* D34T, with 98.83 % of similarities, corresponding to 16 nucleotide differences). According to the phylogenetic tree generated using the 16S rRNA gene sequences, strain S26 is grouped with *Saccharothrix texasensis* NRRL B-16134 (98.76% of similarities corresponding to 17 nucleotide differences) that relationship was supported by two of the three tested algorithms however with a low bootstrap value (Figure 5).

## 3. Materials and Methods

### 3.1. Isolation of Bacterial Strain

**Figure 6:**
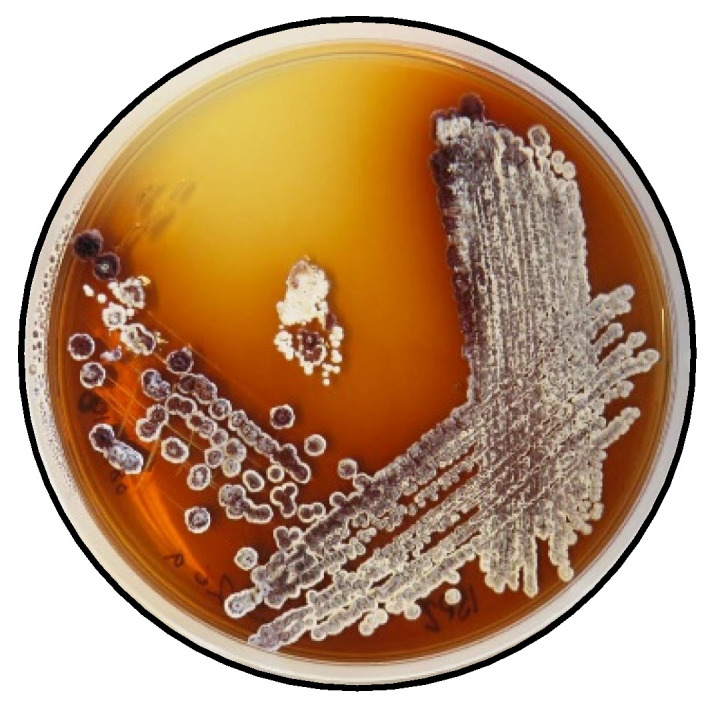
*Saccharothrix* sp. *S26* on ISP2 agar.

Strain *Saccharothrix* sp. S26 was isolated from the rhizosphere of *Lupinus oreophilus* plants growing at 3646 m above sea level in the Atacama Desert in Chile (23°37′06″ S/67°50′56″ W). The rhizosphere sample was collected by JFC and VR on 03.12.2017. The procedure used for the isolations was as follows: a sample of the soil (0.1 g) was sprinkled over plates of starch casein agar (SCA) (composition per liter: soluble starch: 10 g, K_2_HPO_4_: 2 g, KNO_3_: 2 g, NaCl: 2 g, casein: 0.3 g, MgSO_4_·7H_2_O: 0.05 g, CaCO_3_: 0.02 g, FeSO_4_·7H_2_O: 0.01 g, agar: 15 g; pH 7.0) supplemented with cycloheximide (25 μg/mL), nalidixic acid (20 μg/mL) and nystatin (25 μg/mL). The plates were incubated at 30 °C for 21 days. Colonies with Actinomycetes morphology were transferred to fresh SCA plates and incubated at 30 °C for 14 days. Axenic cultures for the strain *Saccharothrix* sp. S26 and other isolates were obtained. All isolates were maintained on ISP 2 agar (Figure 6) [20] and in 20% *v*/*v* glycerol at −80 °C for long-term preservation.

### 3.2. Isolation and Characterisation of the Compound

*Saccharothrix* sp. S26 was cultured in 6 L of tryptic soy broth (TSB- 30 g/L) at room temperature for three weeks, which resulted in a deep red colour consistent with the agar plate permeating throughout the culture medium. The secondary metabolites produced were adsorbed onto diaion HP-20 resin added at 60 g/L and after which the resin was extracted exhaustively with methanol which was evaporated under reduced pressure yielding about 10 g of extract. The extract was dissolved in milliQ water and partitioned thrice with dichloromethane (DCM), which revealed a fraction weighing about 1.84 g. 

The DCM fraction was dissolved in 20 mL of 80% methanol and was subjected to flash chromatography with a gradient starting from 80% methanol and ending with an isocratic period of 15 min of 100% methanol at a flow rate of 12 mL/min using a Buchi Reveleris (Suffolk, UK) X2 flash chromatography system with a C-18 column (Flashpure ecoflex 80 g, particle size: 50 µm, pore size: 92–108 Å) as the stationary phase, which yielded four fractions. One of the fractions, weighing 380 mg, was subjected to high performance liquid chromatography (HPLC) using an Agilent (Cheshire, UK) 1100 series binary pump and 1100 series diode array detector (DAD) with a Sunfire C18(10 µm, 10 mm × 250 mm) column at 2 mL/min, resulting in Mutactimycin AP (**1**) being isolated as a brick-red solid weighing 150 mg. LC-MS was performed using an Agilent 1290 infinity UHPLC and a Phenomenex Kinetex XB-C18 (2.6 µM, 100 × 2.1 mm^2^) column with mobile phases as 5% acetonitrile + 0.1% formic acid and 94.9% water to 100% acetonitrile + 0.1% formic acid. The MS was performed using a Bruker Maxis Q-tof II (Coventry, UK) where the mass range was set from 100–2000 and capillary voltage was set to 4.5 kV, the nebulizer gas set to 4 bar, the dry gas set to 9 L/min, and the dry temperature was set to 220 °C. The MS/MS experiments were conducted under Auto MS/MS scan mode with a step collision energy from 80–200%. The Bruker Avance (Coventry, UK) III HD 400 MHz system with a liquid helium cooled Prodigy cryo-probe was used to measure the NMR spectrum at 25 °C. 

### 3.3. Conformational Analysis of **1**

Structures for the possible stereoisomers of **1** were built using the Avogadro software package (version 1.2) [21]. The lowest energy conformers of each were generated with the iterative meta-dynamics with the genetic crossing (iMTD-GC) method implemented in the CREST software package (version 2.11.2) interfaced to the extended tight-binding semi-empirical electronic structure code xTB (version 6.4.1) [22,23]. In all CREST calculations, the self-consistent charge tight-binding semi-empirical quantum chemical method GFN2-xTB was used [24]. This method is expected to be accurate for the studied structures and includes the recent D4 density-dependent dispersion correction which provides improved descriptions of non-covalent interactions that are likely to be important in these systems [24,25,26,27,28]. The adaptive linearized Poisson-Boltzmann (ALPB) continuum solvent model for methanol was used for all xTB calculations [29].

The lowest energy conformations obtained from the CREST calculations were then used as an input for re-optimizations at the density functional theory (DFT) level using the Orca electronic structure package (version 5.0.3) [30,31]. The dispersion-corrected r2SCAN-D4 meta-GGA DF approximation was used [31,32,33] in combination with the triple-ζ Def2-TZVP [34] basis set. Default integration grids were used and the resolution of the identity (RI) approximation for Coulomb interactions was employed with the Def2/J auxiliary basis set [35]. Solvent (methanol) effects were modelled using the conductor-like polarizable continuum model [36,37].

### 3.4. Antimicrobial Assays

The antimicrobial activity of compound **1** was evaluated against ESKAPE Pathogens (i.e., *Enterococcus faecium* (DSM17050), *Staphylococcus aureus* (DSM2569), *Klebsiella pneumoniae* (DSM681), *Acinetobacter baumannii* (DSM30008), *Pseudomonas aeruginosa* (1117), and *Enterobacter cloacae subsp. Cloacae* (DSM30054) procured from the DSMZ-German Collection of Microorganisms and Cell Cultures GmbH using the disc diffusion method as previously described [38], with slight modifications. Filter paper disks containing oxytetracycline (30 μg) were used as positive controls and DMSO as the negative control. The MIC against test organisms was determined by the broth dilution method as previously described [38], with slight modifications. Test organisms were cultured in sterile Mueller Hinton broth in test tubes with serially diluted compound **1** added (in DMSO). By adding sterile broth to the microbial cultures, a total volume of 2 mL was obtained. The MIC was defined as the lowest concentration of the dilution that showed no apparent growth (without turbidity) after 18 to 24 h of incubation at 28 °C. The MBC (minimum bactericidal concentration) was determined by plating the dilution representing the MIC and two of the more concentrated test product dilutions on LB agar as previously described [39]. An aliquot of the positive control was plated and used to establish a baseline concentration of the microorganism used.

The antimicrobial activity of compound **1** was evaluated against bovine mastitis bacterial Pathogens (i.e., *Enterococcus pseudoavium* NCIMB13084, *Escherichia coli* NCIMB701266, *Klebsiela oxytoca* NCIMB701361, *Staphylococcus aureus subsp. Aureus* NCIMB701494, and *Streptococcus bovis* NCIMB702087) procured from NCIMB Ltd., Aberdeen, UK, using the disc diffusion method as previously described, with slight modifications. Filter paper disks containing oxolinic acid (2 μg) were used as positive controls and DMSO was used as the negative control. The MIC and MBC were measured using the same protocol as mentioned above. All assays were only performed once.

### 3.5. Phylogenetic Analyses

The genomic DNA of *Saccharothrix sp. S26* was extracted with the QIAGEN DNeasy UltraClean Microbial Kit (Cat. No. 12224-50) following the manufacturer’s instructions.

Amplification of 16S rRNA was mediated by PCR using Phusion plus the green PCR master kit (Cat. No. F632S) with the universal primers 27F and 1492R. PCR conditions were: initial denaturation at 98 °C for 2 min; 30 cycles of denaturation at 98 °C for 15 s, annealing at 60 °C for 15 s and extension at 72 °C for 45 s; and final extension at 72 °C for 5 min.

The calculation of pairwise 16S rRNA gene sequence similarities to identify phylogenetic neighbors was realized using the EZBioCloud [40] MUSCLE algorithm from MEGA X [41] was used to align the 16S rRNA sequences. MEGA X software was used to generate the phylogenetic trees using neighbor-joining, maximum-likelihood, and maximum-parsimony algorithms based on 1000 bootstrap replicates. The trees were rooted using the 16S rRNA gene sequence of *Umezawaea tangerina* MK27-91F and *Crossiella cryophila* NRRL B-16238.

## 4. Conclusions

A new compound, mutactimycin AP (**1**), belonging to the mutactimycin family, a group of anthracycline-type compounds, has been isolated from a *Saccharothrix* found in the Atacama Desert. The structure of the compound was determined using HR-ESI-LCMS and NMR spectrometry. This is the first mutactimycin which does not have an oxygen at position 11. The stereochemistry of the compound was confirmed using ^13^C chemical shifts and through-space NMR spectrometry, after which it was validated using molecular modelling. 

The compound showed antibiotic activity against methicillin resistant *Staphylococcus aureus* when tested against the ESKAPE pathogens. This is consistent with previously isolated mutactimycins and anthracyclines in general therefore mutactimycin AP can be considered a potential hit in battling this group of multidrug resistant bacteria. Compound **1** also showed activity against *Enterococcus pseudoavium* along with *Staphylococcus aureus subsp. aureus*. These strains are among a panel of pathogenic strains, against which the compound was tested, which are associated with bovine mastitis, a highly prevalent and complex disease among dairy cattle worldwide. Thus, **1** can be considered as a possible lead candidate for battling this devastating infection. 

The strain, upon analysis of its genome, has been found to have an interesting phylogenetic tree with it having a close relationship with other extreme environment bacteria belonging to the genus *Saccharothrix*.

Actinomycetes have been hailed as an inexhaustible source of antibiotics with a fascinating and diverse range of structural moieties. In recent decades there has been an increase in the emergence of multidrug resistant strains across the world due to which there is a need for the development of novel anti-bacterial agents to combat them. Natural products provide excellent structural diversity compared to standard ways of discovering new potential lead molecules, therefore isolating them is a worthwhile pursuit. 

## Figures and Tables

**Table 1:** NMR data of compound **1** in DMSO-d6 at 400 MHz with a (*) denoting a weaker correlation. The values in bold belong to Mutactimycin E (**4)** [14].

Position	*δ*^13^C	*δ*^1^H (Multiplicity, *J* in Hz)	COSY (H→H)	HMBC (C→H)	*δ*^13^C [14]	*δ*^1^H (Multiplicity, *J* in Hz) [14]
1	118.8	7.87 (d, 7.9)	2		**122.6**	**7.98 (d, 7.8)**
2	137.2	7.74 (d, 7.9)	1	3 Me	**137.0**	**7.78 (d, 7.9)**
3	141.1	-	-	3 Me*, 11	**141.4**	
3 Me	16.3	2.35 (s)	-	2	**16.3**	**2.37 (s)**
4	158.9	-	-	2*, 4 OMe, 3 Me	**159.0**	
4a	124.7	-	-	11, 3 Me*	**125.0**	
4 OMe	60.6	3.82 (s)	-		**60.7**	**3.82 (s)**
5	188.3	-	-	1*	**186.5**	
5a	114.4	-	-	1	**110.0**	
6	161.7	-	-	1*, 7	**157.8**	
6a	131.0	-	-		**135.7**	
7	72.2	4.89 (t)	8	1′	**74.9**	**4.90 (d, 3.8)**
8	43.1	H_A_:2.12 (dd, 13.7, 6.4) H_B_:1.97 (dd, 5.19, 13.89)	7	9Me	**73.0**	**3.67 (d, 4.1)**
9	67.5	-		7, 10*, 8*, 9 Me*	**70.1**	
9 Me	28.7	1.29 (s)	-		**26.6**	**1.32 (s)**
10	44.3	H_A_:2.94 (d, 15.7), H_B_:2.75 (d, 17.01)	10	11, 9 Me,	**33.8**	**2.77 (d, 18.1)** **2.63 (d, 18.2)**
10a	146.0	-	-	1*, 7*, 10*	**137.7**	
11	122.9	7.35 (s)	-	12	**155.0**	
11a	131.2	-	-	1, 7, 10*, 8*	**110.7**	
12	181.5	-	-	1, 11	**185.7**	
12a	133.2	-	-	2, 3 Me*	**133.1**	
1′	103.5	5.11 (s)	2′	7	**103.7**	**5.19 (br s)**
2′	66.3	3.85 (s)	3′,1′	1′, 3′*	**66.3**	**3.97 (m)**
3′	80.7	3.00 (dd, 9.5, 2.96)	4′,2′	1′, 4′*, 3′ OMe	**80.2**	**3.29 (dd, 9.3, 3.3)**
3′OMe	56.2	3.22 (s)	-	3′	**56.1**	**3.31 (dd, 9.3, 9.3)**
4′	70.6	3.32 (t, 9.3)	5′,3′	3′ OMe*, 10*, 6′	**70.7**	**3.26 (s)**
5′	69.3	3.63 (dt, 9.3, 5.89)	4′,6′	1′, 3′	**69.2**	**3.80 (dq, 9.2, 6.2)**
6′	17.9	1.21 (d, 6.1)	5′	4′	**17.9**	**1.19 (d, 6.2)**
6-OH		13.5 (br s)	-	-		

**Table 2:** Bioactivity data of **1** against strains associated with bovine mastitis.

Bacterial Strain	Inhibition Zones (mm)		MIC of 1 (μg/mL)
	Compound 1	Oxalonic Acid	
*Enterococcus pseudoavium* NCIMB 13084	18	17	12.5
*Staphylococcus aureus subsp. Aureus* NCIMB 701494	12	22	25

## Data Availability

Not applicable.

## Supplementary Materials

The following supporting information can be downloaded at: www.mdpi.com/article/10.3390/molecules27217185/s1, Figure S1: Annotated mass spectrum of compound **1**; Figure S2: Annotated UV spectrum of compound **1**: Figure S3: Annotated 1H NMR spectrum of compound **1** in DMSO-d6 at 400 MHz; Figure S4: Annotated 13C NMR spectrum of compound **1** in DMSO-d6 at 400 MHz; Figure S5: HSQC spectrum of compound **1** in DMSO-d6 at 400 MHz; Figure S6: HMBC spectrum of compound **1** in DMSO-d6; Figure S7: COSY spectrum of compound **1** in DMSO-d6; Figure S8: 2D ROESY spectrum of compound **1** in DMSO-d6; Figure S9: 2D ROESY spectrum of compound **1** in CD_3_OD at 400 MHz; Table S1: comparison of distances between H7, H3′ and H9Me measured with all 4 diastereomers of **1**; Figure S10–S12: distances between H7, H3′ and H9Me measured with the 3 remaining diastereomers; Figure S13: Disc diffusion assay results of compound 1 against (a) *Enterococcus pseudoavium* NCIMB 13084 (b) *Staphylococcus aureus subsp. aureus* NCIMB 701494 (c) Methicillin resistant *Staphylococcus aureus* DSMZ 2569.

## Sample Availability

Samples of the compounds are available from the authors.

## References

[1] Barka E.A., Vatsa P., Sanchez L., Gaveau-Vaillant N., Jacquard C., Klenk H.-P., Clément C., Ouhdouch Y., van Wezel G.P. (2016). Taxonomy, Physiology, and Natural Products of Actinobacteria. Microbiol. Mol. Biol. Rev..

[2] de Lima Procópio R.E., da Silva I.R., Martins M.K., de Azevedo J.L., de Araújo J.M. (2012). Antibiotics produced by Streptomyces. Braz. J. Infect. Dis..

[3] Watve M.G., Tickoo R., Jog M.M., Bhole B.D. (2001). How many antibiotics are produced by the genus Streptomyces?. Arch. Microbiol..

[4] Rateb M.E., Houssen W.E., Arnold M., Abdelrahman M.H., Deng H., Harrison W.T.A., Okoro C.K., Asenjo J.A., Andrews B.A., Ferguson G. (2011). Chaxamycins A-D, bioactive ansamycins from a hyper-arid desert Streptomyces sp.. J. Nat. Prod..

[5] Schulz D., Beese P., Ohlendorf B., Erhard A., Zinecker H., Dorador C., Imhoff J.F. (2011). Abenquines A–D: Aminoquinone derivatives produced by Streptomyces sp. strain DB634. J. Antibiot..

[6] Rateb M.E., Ebel R., Jaspars M. (2018). Natural product diversity of actinobacteria in the Atacama Desert *Antonie van Leeuwenhoek*. Int. J. Gen. Mol. Microbiol..

[7] Strobel T., Schmidt Y., Linnenbrink A., Luzhetskyy A., Luzhetska M., Taguchi T., Brötz E., Paululat T., Stasevych M., Stanko O. (2013). Tracking Down Biotransformation to the Genetic Level: Identification of a Highly Flexible Glycosyltransferase from Saccharothrix espanaensis. Appl. Environ. Microbiol..

[8] Ohuchi T., Ikeda-Araki A., Watanabe-Sakamoto A., Kojiri K., Nagashima M., Okanishi M., Suda H. (2000). Cloning and expression of a gene encoding N-glycosyltransferase (ngt) from Saccarothrix aerocolonigenes ATCC39243. J. Antibiot..

[9] Mikami Y., Yazawa K., Ohashi S., Maeda A., Akao M., Ishibashi M., Kobayashi J., Yamazaki C. (1992). SO-75R1, A new mutactimycin derivative produced by Nocardia brasiliensis. J. Antibiot..

[10] Zitouni A., Mathieu F., Coppel Y., Pont F., Sabaou N., Lebrihi A. (2004). Mutactimycin PR, a new anthracycline antibiotic from Saccharothrix sp. SA 103. II. Physico-chemical properties and structure elucidation. J. Antibiot..

[11] Wadler S., Fuks J.Z., Wiernik P.H. (1986). Phase I and II Agents in Cancer Therapy: I. Anthracyclines and Related Compounds. J. Clin. Pharmacol..

[12] Minotti G., Menna P., Salvatorelli E., Cairo G., Gianni L. (2004). Anthracyclines: Molecular advances and pharmacologic developments in antitumor activity and cardiotoxicity. Pharmacol. Rev..

[13] Weiss R.B. (1992). The anthracyclines: Will we ever find a better doxorubicin?. Semin. Oncol..

[14] Hopp D.C., Rabenstein J., Rhea J., Smith C., Romari K., Clarke M., Francis L., Irigoyen M., Milanowski D., Luche M. (2008). Mutactimycin E, a New Anthracycline Antibiotic with Gram-positive Activity. J. Antibiot..

[15] Speitling M., Nattewan P., Yazawa K., Mikami Y., Grün-Wollny I., Ritzau M., Laatsch H., Gräfe U. (1998). Demethyl mutactimycins, new anthracycline antibiotics from Nocardia and Streptomyces strains. J. Antibiot..

[16] Stan D., Enciu A.-M., Mateescu A.L., Ion A.C., Brezeanu A.C., Tanase C. (2021). Natural Compounds With Antimicrobial and Antiviral Effect and Nanocarriers Used for Their Transportation. Front. Pharmacol..

[17] Alonso M.M., Salazar J.C.L., Robles S.O., Guerrero I.C., García F.L., Marrero J.G. (2020). In vitro antimicrobial activity of mexican plants on bovine mastitis bacteria: Preliminary studies. Biosci. J..

[18] Nett M., Ikeda H., Moore B.S. (2009). Genomic basis for natural product biosynthetic diversity in the actinomycetes. Nat. Prod. Rep..

[19] Takahashi Y., Nakashima T. (2018). Actinomycetes, an Inexhaustible Source of Naturally Occurring Antibiotics. Antibiotics.

[20] Shirling E.B., Gottlieb D. (1966). Methods for characterization of Streptomyces species1. Int. J. Syst. Evol. Microbiol..

[21] Hanwell M.D., Curtis D.E., Lonie D.C., Vandermeerschd T., Zurek E., Hutchison G.R. (2012). Avogadro: An advanced semantic chemical editor, visualization, and analysis platform. J. Cheminform..

[22] Pracht P., Bohle F., Grimme S. (2020). Automated exploration of the low-energy chemical space with fast quantum chemical methods. Phys. Chem. Chem. Phys..

[23] Bannwarth C., Caldeweyher E., Ehlert S., Hansen A., Pracht P., Seibert J., Spicher S., Grimme S. (2021). Extended tight-binding quantum chemistry methods. Wiley Interdiscip. Rev. Comput. Mol. Sci..

[24] Bannwarth C., Ehlert S., Grimme S. (2019). GFN2-xTB—An Accurate and Broadly Parametrized Self-Consistent Tight-Binding Quantum Chemical Method with Multipole Electrostatics and Density-Dependent Dispersion Contributions. J. Chem. Theory Comput..

[25] Caldeweyher E., Bannwarth C., Grimme S. (2017). Extension of the D3 dispersion coefficient model. J. Chem. Phys..

[26] Caldeweyher E., Ehlert S., Hansen A., Neugebauer H., Spicher S., Bannwarth C., Grimme S. (2019). A generally applicable atomic-charge dependent London dispersion correction. J. Chem. Phys..

[27] Caldeweyher E., Mewes J.-M., Ehlert S., Grimme S. (2020). Extension and evaluation of the D4 London-dispersion model for periodic systems. Phys. Chem. Chem. Phys..

[28] Ehlert S., Stahn M., Spicher S., Grimme S. (2021). Robust and Efficient Implicit Solvation Model for Fast Semiempirical Methods. J. Chem. Theory Comput..

[29] Neese F. (2012). The ORCA program system. WIREs Comput. Mol. Sci..

[30] Neese F. (2018). Software update: The ORCA program system, version 4.0. WIREs Comput. Mol. Sci..

[31] Furness J.W., Kaplan A.D., Ning J., Perdew J.P., Sun J. (2020). Accurate and Numerically Efficient r^2^ SCAN Meta-Generalized Gradient Approximation. J. Phys. Chem. Lett..

[32] Ehlert S., Huniar U., Ning J., Furness J.W., Sun J., Kaplan A.D., Perdew J.P., Brandenburg J.G. (2021). r^2^SCAN-D4: Dispersion corrected meta-generalized gradient approximation for general chemical applications. J. Chem. Phys..

[33] Weigend F., Ahlrichs R. (2005). Balanced basis sets of split valence, triple zeta valence and quadruple zeta valence quality for H to Rn: Design and assessment of accuracy. Phys. Chem. Chem. Phys..

[34] Weigend F. (2006). Accurate Coulomb-fitting basis sets for H to Rn. Phys. Chem. Chem. Phys..

[35] Tomasi J., Mennucci B., Cammi R. (2005). Quantum Mechanical Continuum Solvation Models. Chem. Rev..

[36] Mennucci B. (2012). Polarizable continuum model. WIREs Comput. Mol. Sci..

[37] Siddharth S., Vittal R.R. (2018). Evaluation of Antimicrobial, Enzyme Inhibitory, Antioxidant and Cytotoxic Activities of Partially Purified Volatile Metabolites of Marine Streptomyces sp.S2A. Microorganisms.

[38] Siddharth S., Vittal R.R. (2019). Isolation, characterization, and structural elucidation of 4-methoxyacetanilide from marine actinobacteria Streptomyces sp. SCA29 and evaluation of its enzyme inhibitory, antibacterial, and cytotoxic potential. Arch. Microbiol..

[39] Tedesco P., Maida I., Esposito F.P., Tortorella E., Subko K., Ezeofor C.C., Zhang Y., Tabudravu J., Jaspars M., Fani R. (2016). Antimicrobial Activity of Monoramnholipids Produced by Bacterial Strains Isolated from the Ross Sea (Antarctica). Mar. Drugs.

[40] Yoon S.-H., Ha S.-M., Kwon S., Lim J., Kim Y., Seo H., Chun J. (2017). Introducing EzBioCloud: A taxonomically united database of 16S rRNA gene sequences and whole-genome assemblies. Int. J. Syst. Evol. Microbiol..

[41] Kumar S., Stecher G., Li M., Knyaz C., Tamura K. (2018). MEGA X: Molecular Evolutionary Genetics Analysis across Computing Platforms. Mol. Biol. Evol..

