# Supplementary material for: Mutactimycin AP, a New Mutactimycin Isolated from an Actinobacteria from the Atacama Desert
Source: Molecules. 2022 Oct 24;27(21):7185. doi: 10.3390/molecules27217185 (PMC9656151; doi:10.3390/molecules27217185)
Supplement: Supplementary file 1 [file molecules-27-07185-s001.zip › molecules-1964943-supplementary.pdf]

molecules

MDPI

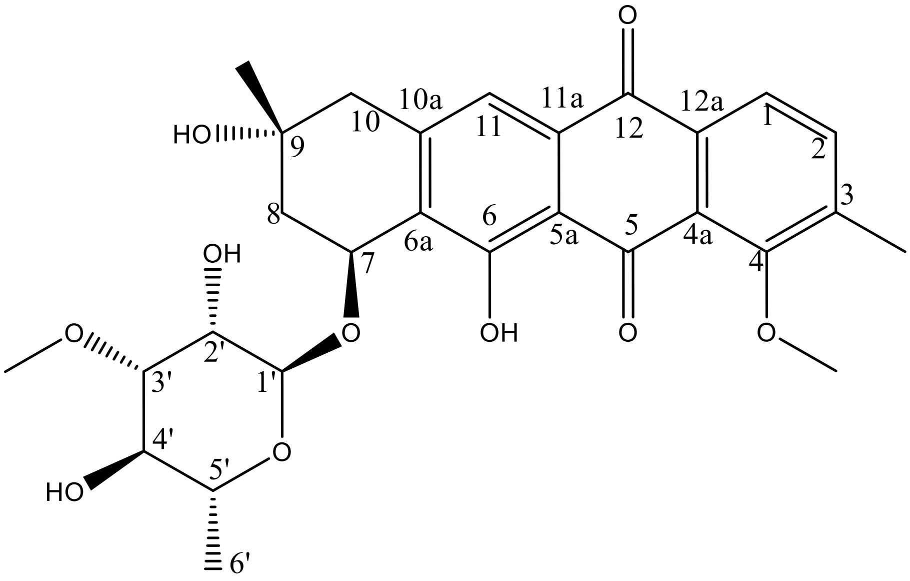

Structure of compound **1**

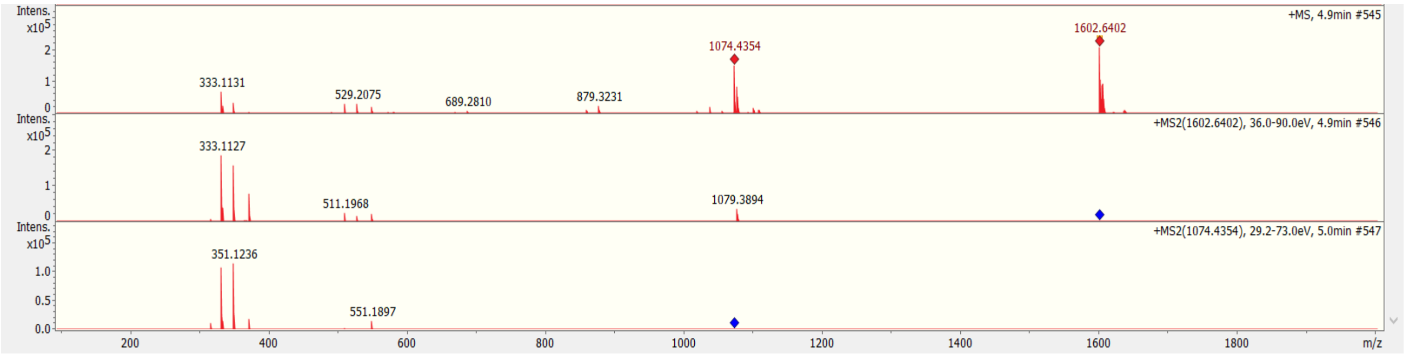

Figure S1: Mass spectrum of compound **1**

$[M+H]^+$ appears at m/z 529.2075 with formula $C_{28}H_{33}O_{10}$ (error: -1.2 ppm msigma: 7.2)

The $[\text{aglycone} + H]^+$ appears at m/z 351.1236

The m/z appearing at 551.1897 corresponds to $[M+Na]^+$

The m/z appearing at 1074.4354 corresponds to $[2M+NH_4]^+$

The m/z appearing at 1079.3894 corresponds to $[2M+Na]^+$

The m/z appearing at 1602.6402 corresponds to $[3M+Na]^+$

molecules

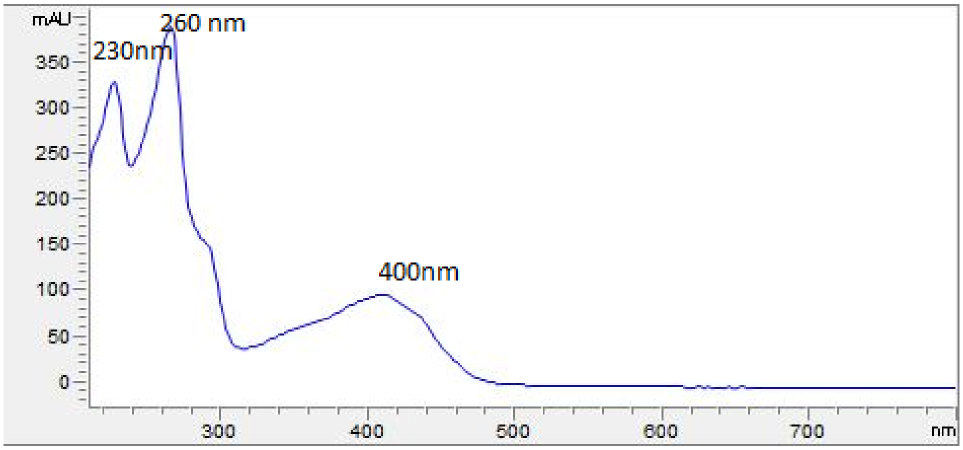

Figure S2: Annotated UV spectrum of compound **1**

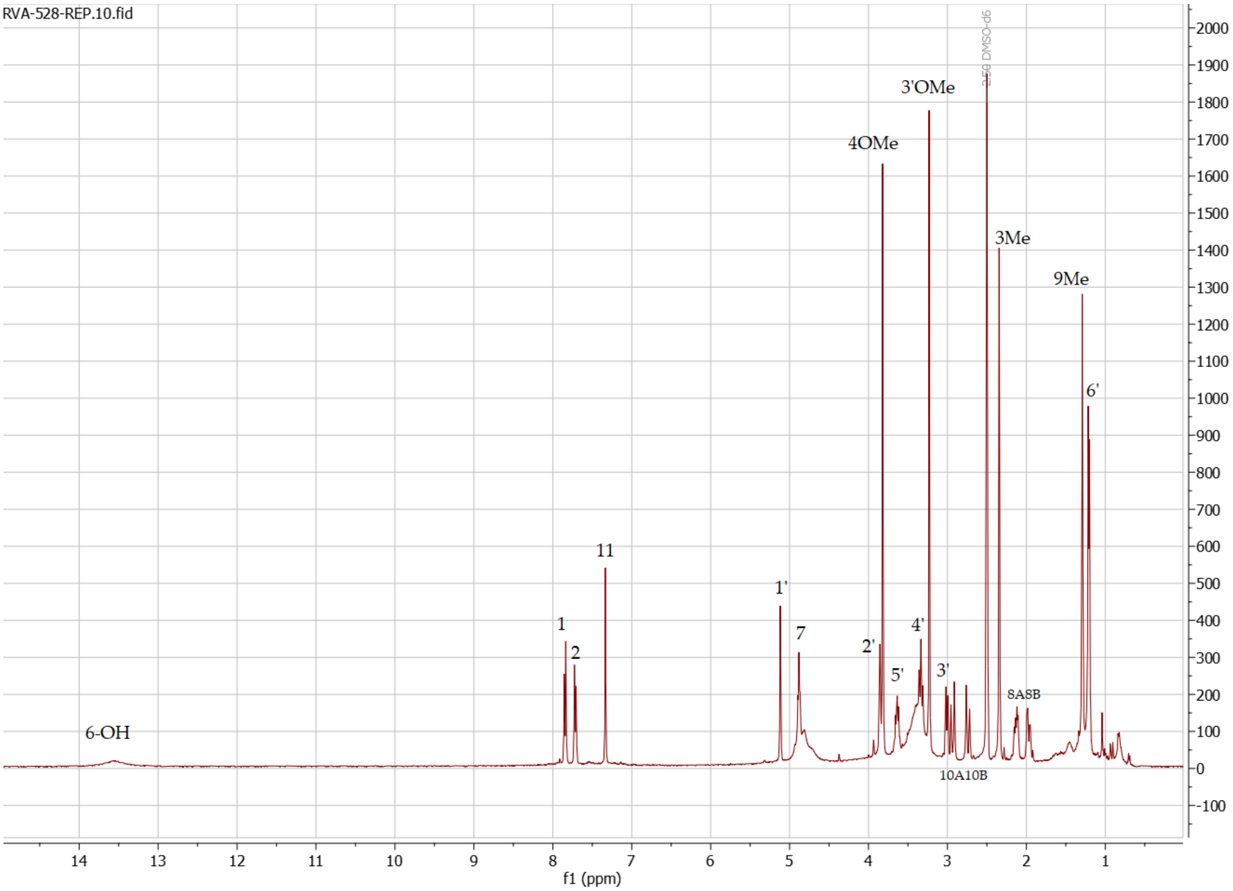

Figure S3: Annotated 1H NMR spectrum of compound **1** in DMSO-d6 at 400MHz

molecules

MDPI

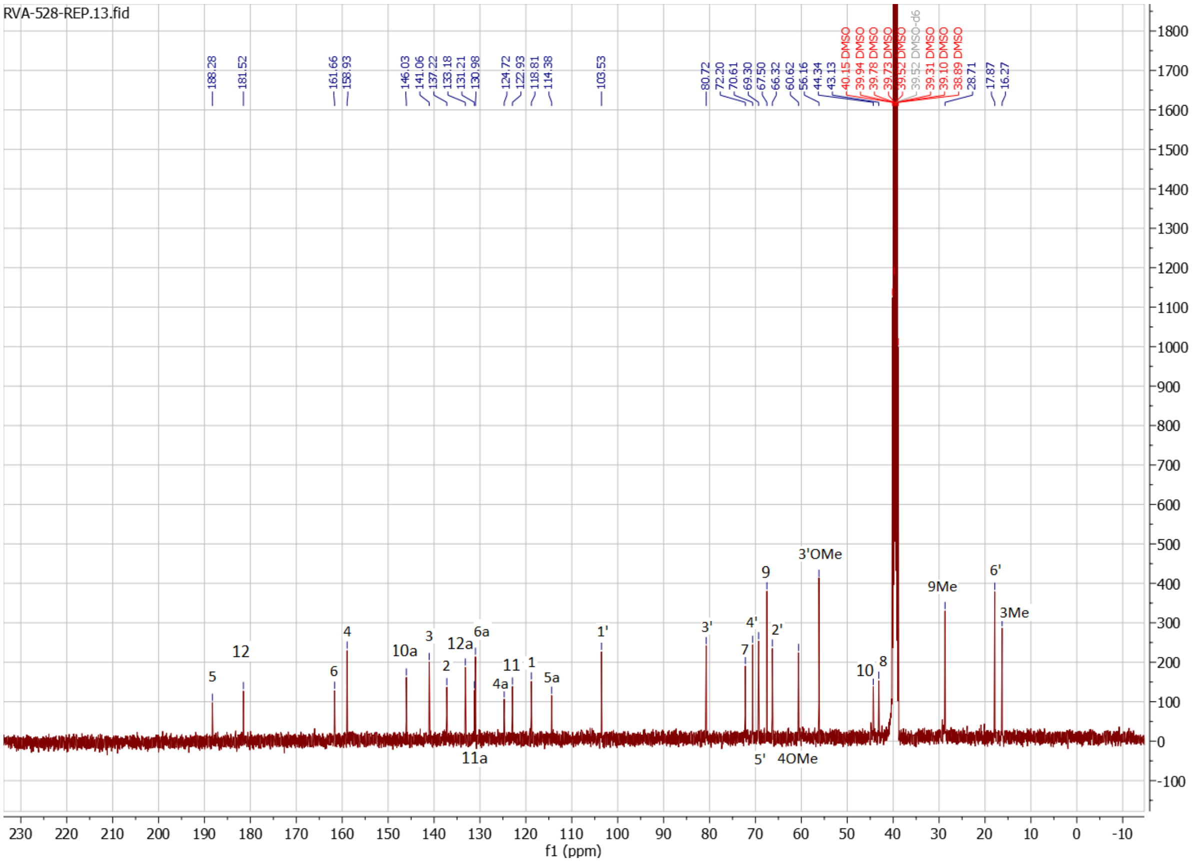

Figure S4: Annotated 13C NMR spectrum of compound **1** in DMSO-d6 at 400MHz

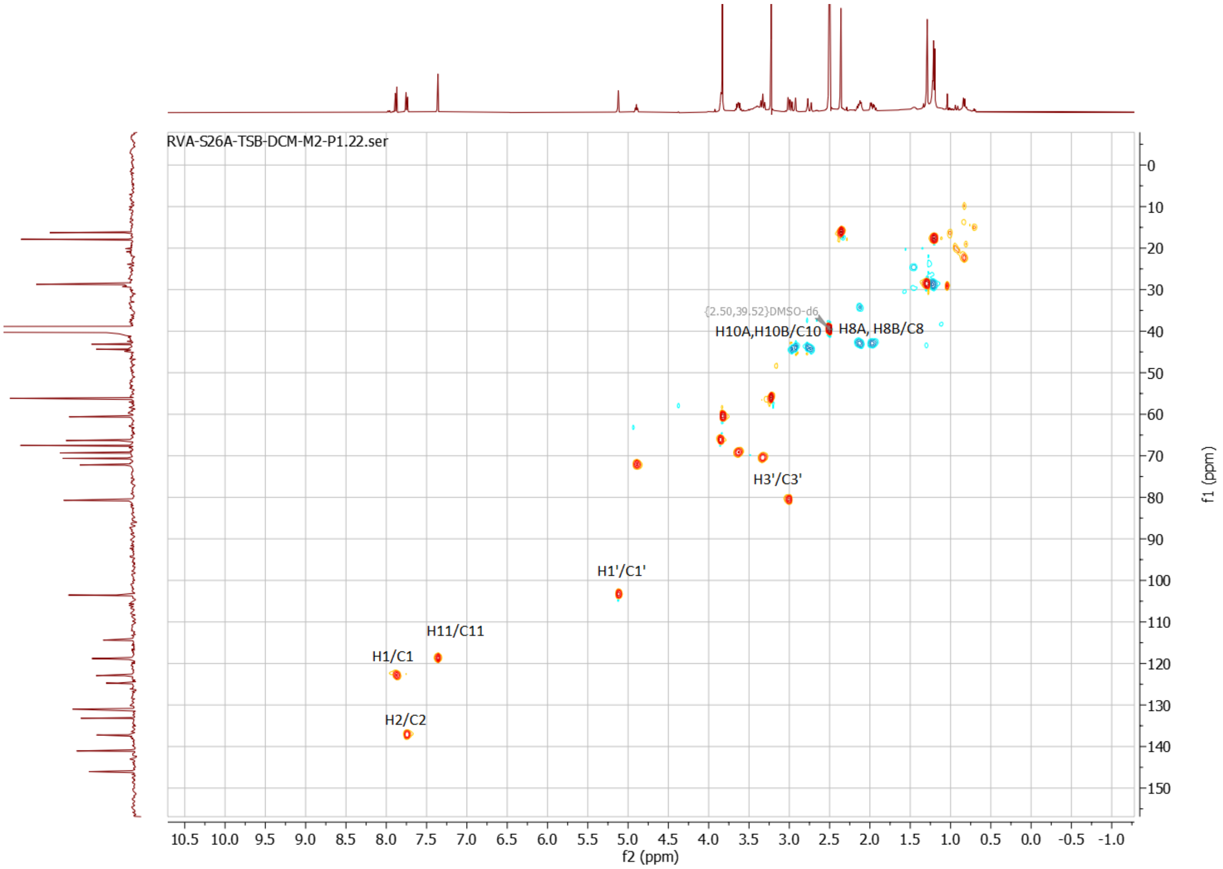

Figure S5: HSQC spectrum of compound **1** in DMSO-d6 at 400 MHz with Key correlations annotated

molecules

MDPI

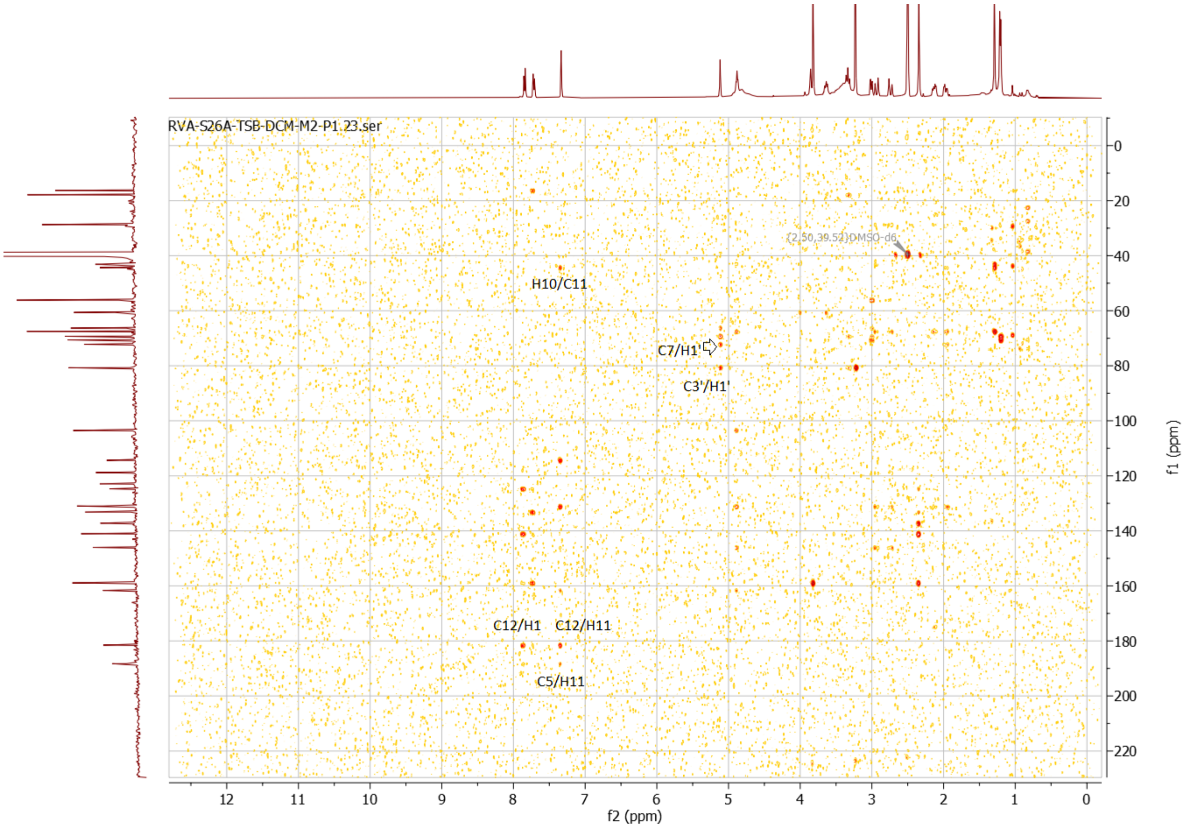

Figure S6: HMBC spectrum of compound **1** in DMSO at 400 MHz with key correlations annotated.

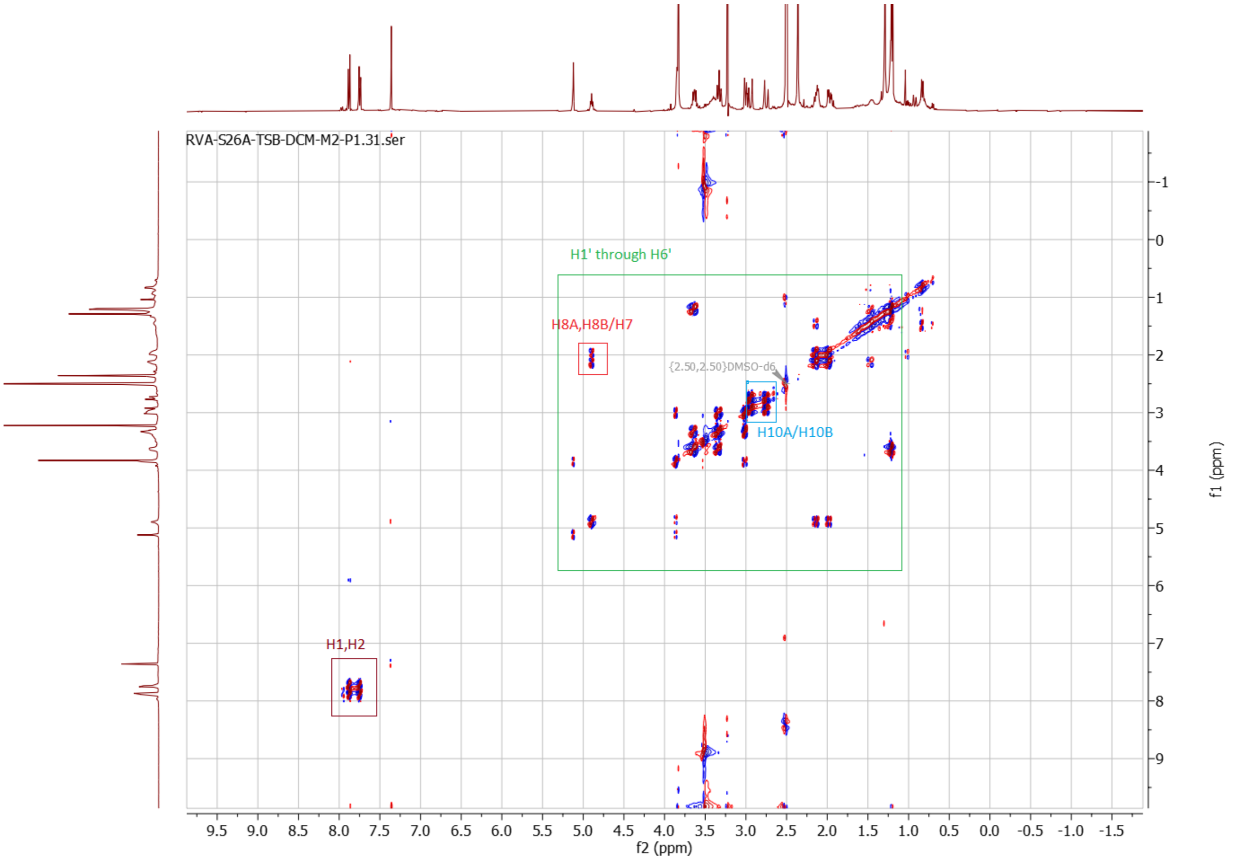

Figure S7: DQF COSY of compound **1** in DMSO at 400 MHz with key correlations annotated

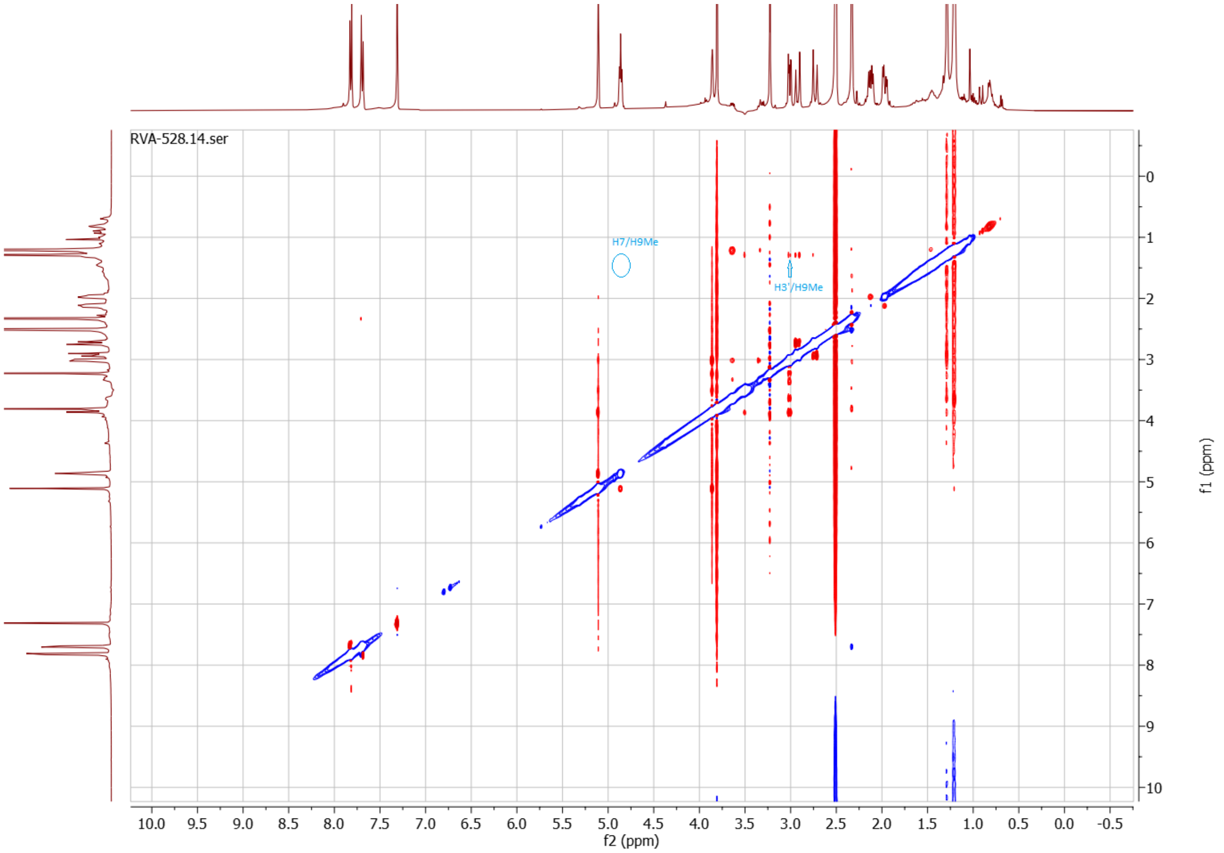

Figure S8: ROESY spectrum of compound **1** in DMSO-d6 at 400 MHz with the correlation between H9Me and H3’ highlighted and the lack of correlation between H7 and H9Me at the same level also highlighted.

molecules MDPI

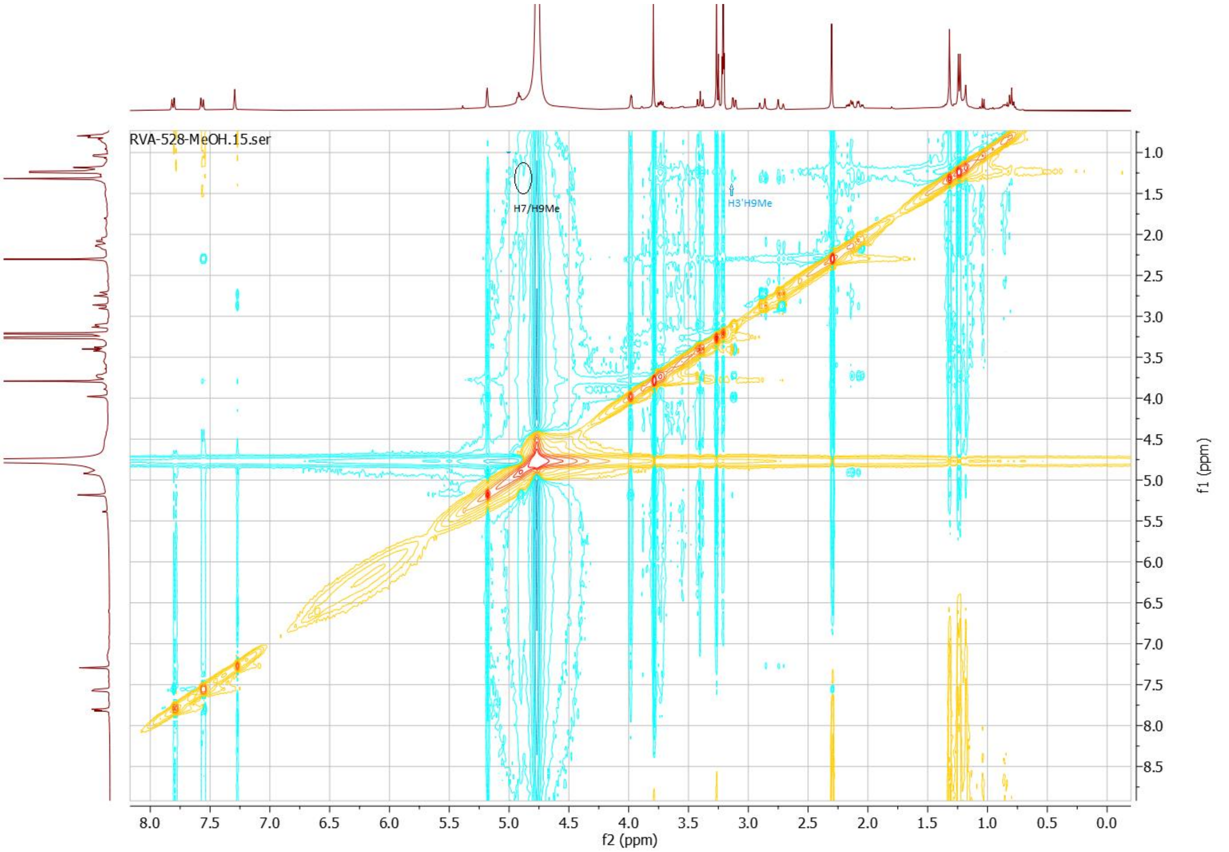

Figure S9: ROESY spectrum of compound **1** in CD_3OD at 400 MHz with the correlation between H9Me and H3' highlighted and the lack of correlation between H7 and H9Me at the same level also highlighted

molecules MDPI

Diastereomer 7S9R	Diastereomer 7R9R	Diastereomer 7R9S	Diastereomer 7S9S
Distances between **H9Me** and			
**H7**: 3.9, 4.4, 5.2<br>GM = 4.5	**H7**: 4.8, 5.5, 4.5<br>GM = 4.9	**H7**: 4.5, 5.5,5.8<br>GM = 5.2	**H7**: 4.9, 4.8, 5.6<br>GM = 5.1
**H3'**: 3.3, 3.2, 4.5<br>GM = 3.6	**H3'**: 6.4, 5.3, 5.7<br>GM = 5.8	**H3'**:3.6, 5.3, 4.9<br>GM = 4.5	**H3'**: 5.3, 5.9, 6.5<br>GM = 5.9

Table S1: comparison of distances (in Å) and their geometric means between H7, H3' and H9Me measured for all 4 diastereomers of **1**

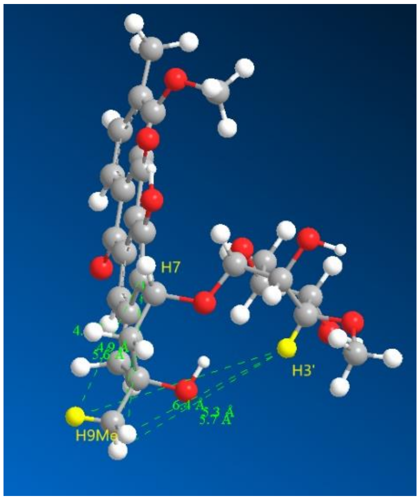

Figure S10: the distances between H7, H3' and H9Me shown where the configuration of position 7 is R and that of position of 9 is R

molecules

MDPI

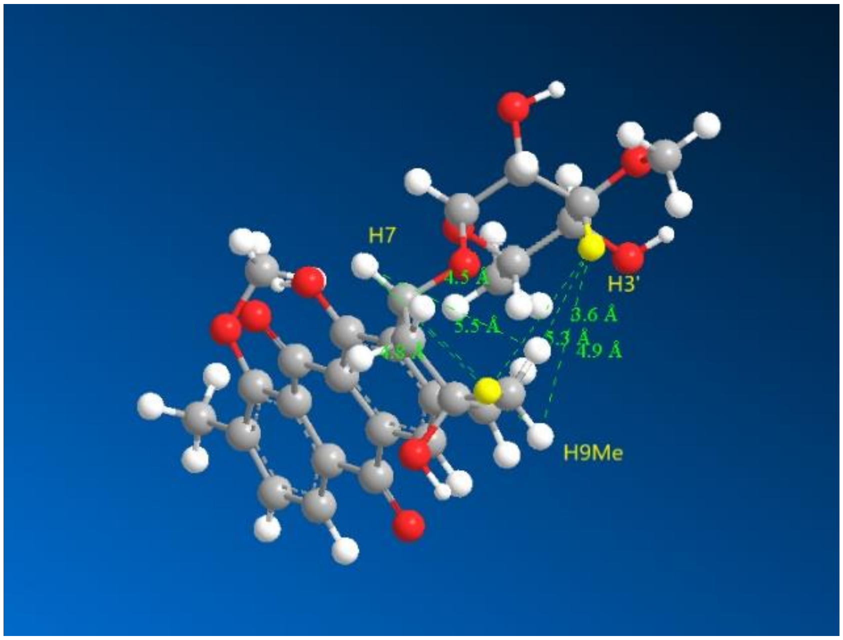

Figure S11: the distances between H7, H3’ and H9Me shown where the configuration of position 7 is R and that of position of 9 is S

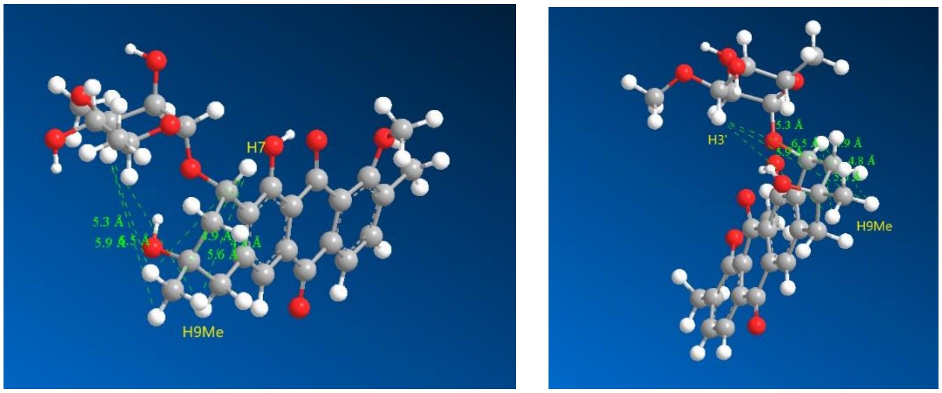

(a) **(b)**

Figure S12: the distances between **(a)** H7 and H9Me and **(b)** H3’ and H9Me shown where the configuration of position 7 is S and that of position of 9 is S

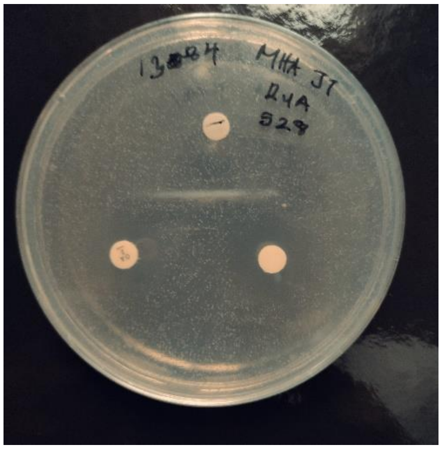

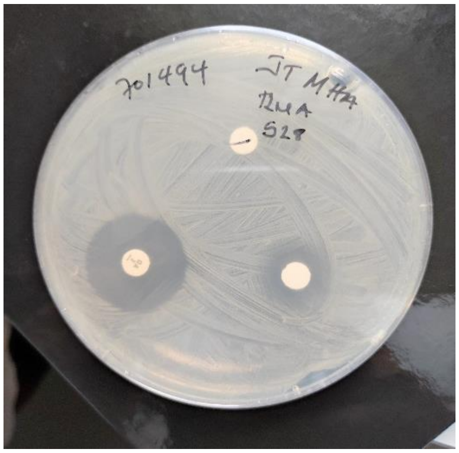

(a) (b)

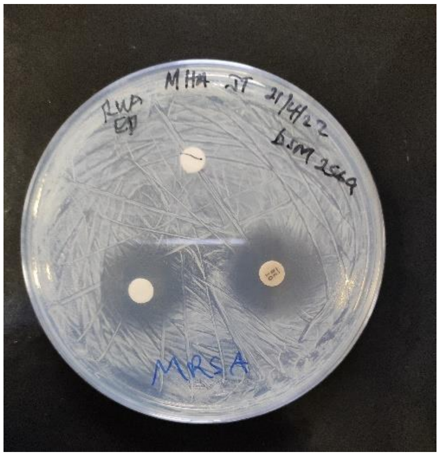

(c)

Figure S13: Disc diffusion assay results of compound **1** against (a) *Enterococcus pseudoavium* NCIMB 13084 (b) *Staphylococcus aureus subsp. aureus* NCIMB 701494 (c) Methicillin resistant *Staphylococcus aureus* DSMZ 2569
